# Cofilin-1, LIMK1 and SSH1 are differentially expressed in locally advanced colorectal cancer and according to consensus molecular subtypes

**DOI:** 10.1186/s12935-021-01770-w

**Published:** 2021-01-22

**Authors:** Annie Cristhine Moraes Sousa-Squiavinato, Renata Ivo Vasconcelos, Adriana Sartorio Gehren, Priscila Valverde Fernandes, Ivanir Martins de Oliveira, Mariana Boroni, Jose Andrés Morgado-Díaz

**Affiliations:** 1 Cellular and Molecular Oncobiology Program, Brazilian National Cancer Institute (INCA), 37 André Cavalcanti Street, 3th Floor, Rio de Janeiro, RJ 20231-050 Brazil; 2 Pathology Division-DIPAT, Brazilian National Cancer Institute (INCA), Rio de Janeiro, Brazil; 3 Bioinformatics and Computational Biology Lab, Division of Experimental and Translational Research, Brazilian National Cancer Institute (INCA), Rio de Janeiro, Brazil

**Keywords:** Cofilin-1, Colorectal cancer, Consensus molecular subtypes, LIMK1, Lymph node metastasis, SSH1

## Abstract

**Background:**

Colorectal cancer (CRC) is among the deadliest cancers, wherein early dissemination of tumor cells, and consequently, metastasis formation, are the main causes of mortality and poor prognosis. Cofilin-1 (CFL-1) and its modulators, LIMK1/SSH1, play key roles in mediating the invasiveness by driving actin cytoskeleton reorganization in various cancer types. However, their clinical significance and prognostic value in CRC has not been fully explored. Here, we evaluated the clinical contribution of these actin regulators according to TNM and consensus molecular subtypes (CMSs) classification.

**Methods:**

CFL-1, LIMK1 and SSH1 mRNA/protein levels were assessed by real-time PCR and immunohistochemical analyses using normal adjacent and tumor tissues obtained from a clinical cohort of CRC patients. The expression levels of these proteins were associated with clinicopathological features by using the chi square test. In addition, using RNA-Seq data of CRC patients from The Cancer Genome Atlas (TCGA) database, we determine how these actin regulators are expressed and distributed according to TNM and CMSs classification. Based on gene expression profiling, Kaplan–Meier survival analysis was used to evaluated overall survival.

**Results:**

Bioinformatic analysis revealed that LIMK1 expression was upregulated in all tumor stages. Patients with high levels of LIMK1 demonstrated significantly lower overall survival rates and exhibited greater lymph node metastatic potential in a clinical cohort. In contrast, CFL-1 and SSH1 have expression downregulated in all tumor stages. However, immunohistochemical analyses showed that patients with high protein levels of CFL-1 and SSH1 exhibited greater lymph node metastatic potential and greater depth of local invasion. In addition, using the CMSs classification to evaluate different biological phenotypes of CRC, we observed that LIMK1 and SSH1 genes are upregulated in immune (CMS1) and mesenchymal (CMS4) subtypes. However, patients with high levels of LIMK1 also demonstrated significantly lower overall survival rates in canonical (CMS2), and metabolic (CMS3) subtypes.

**Conclusions:**

We demonstrated that CFL-1 and its modulators, LIMK1/SSH1, are differentially expressed and associated with lymph node metastasis in CRC. Finally, this expression profile may be useful to predict patients with aggressive signatures, particularly, the immune and mesenchymal subtypes of CRC.

## Background

Colorectal cancer (CRC) it is the third most commonly diagnosed cancer and the second most common cause of cancer related deaths worldwide. Additionally, metastasis is the main cause of death in the majority of patients, and effective biomarkers for risk stratification of early metastatic disease are still required [1]. Decisions regarding clinical treatment are almost exclusively based on clinicopathological parameters, since CRC is a highly heterogeneous disease molecular classifications have been proposed based on the gene expression signatures and tumor biology features, which could lead to improved therapeutic outcomes [2, 3]. Later, one research group performed a more detailed classification of primary colorectal tissue, in which a large dataset (n = 4151 patients) was analyzed and four consensus molecular subtypes (CMS) for CRC were proposed with distinguishing features: CMS1 (microsatellite instability and immunity), CMS2 (epithelial and canonical), CMS3 (epithelial and metabolic), and CMS4 (mesenchymal) [2]. Hence, research has attempted to identify new phenotypic signatures in each subgroup to design an innovative strategy to predict patient stratification and to translate this information into a more homogeneous and effective drug response.

Particularly, the CMS4 displays a lower overall survival rate and relapse-free survival rate, exhibiting a high stromal content and activation of pathways related to epithelial–mesenchymal transition (EMT), mediated by transforming growth factor-β (TGFβ) signaling [2]. EMT is a reversible cellular program in which epithelial cells transiently lose proteins of the junctional complex and acquire mesenchymal proteins which develop quasi-mesenchymal cell states, leading to more aggressive phenotypes [4, 5]. During EMT, the cellular actin cytoskeleton is rearranged and distinct actin structures are stimulated, allowing cell migration and invasion [4]. This process is regulated by various actin-binding proteins that mediate the construction of protrusive and contractile structures, associated with rapid polymerization/depolymerization of filamentous actin (F-actin) [6, 7]. The members of cofilin-1 (CFL-1) and ADF protein family are the most critical components of actin dynamic regulation. CFL-1 promotes actin filament disassembly by severing F-actin and rapidly increasing depolymerization of the filament. CFL-1 activity is tightly regulated by LIM domain kinase 1 (LIMK1) and slingshot protein phosphatase 1 (SSH1) proteins [8]. The precise balance between expression and subcellular localization of CFL-1, LIMK1, and SSH1 is pivotal for small changes in the dynamics of the actin cytoskeleton, and may augment features of tumor aggressiveness, including tumor dissemination [9]. In various cancer types, including gastric, prostate, urothelial, breast, and vulvar, elevated expression of CFL-1 and its regulators, LIMK1/SSH1, have been implicated in tumor progression and aggressiveness, as well as in poor survival rate [10–16]. In the case of CRC, CFL-1 gene expression information is limited and divergent. Some studies have demonstrated elevated gene and protein expression, while others showed decreased expression in relation to normal tissues [17–21].

Since a significant proportion of patients present dissemination of tumor cells with subsequent metastasis growth, both innovative therapies and biomarkers to detect patients with lymph node metastasis potential are needed in CRC treatment. Therefore, an analysis of the profile of gene and protein expression of CFL-1 and its regulators, LIMK1/SSH1, may provide important insights into the local invasion processes of primary tumor cells and could help in disease stratification in routine pathology. In this study, using bioinformatic approaches and a clinical cohort, we aimed to determine the potential role of expression levels, as well as clinical implications, of CFL-1 and its regulators, LIMK1/SSH1, in CRC.

## Materials and methods

### Tumor tissue samples

Primary tumor specimens and their corresponding normal adjacent tissue (at least 5 cm away from the lesion) were obtained from patients with CRC who underwent colectomy and rectosigmoidectomy at the surgical center of the Hospital do Câncer I—Instituto Nacional de Câncer (INCA) (Rio de Janeiro, Brazil). Fresh samples were collected from 2008 to 2018 and were immediately stored in RNA-later (Invitrogen, Carlsbad, CA, USA) at -20 °C for RT-qPCR analysis (n = 38). Formalin-fixed paraffin-embedded CRC tissues (n = 59) from 2009 to 2018 were obtained from the Department of Pathology of INCA and were used for immunohistochemical (IHC) analysis after identification (primary tumors n = 49 and liver metastasis tissue n = 10). None of the patients enrolled had received preoperative radiotherapy or chemotherapy. Details of the clinicopathological description of the patients included in this study are available in Table 1. This study was approved by the Instituto Nacional de Cancer Ethics Research Committee (CEP-INCA), following all relevant guidelines and regulations. Informed consent was obtained from all participants.

**Table 1:** Clinical pathological features of CRC patients with primary tumor

Features	Number of patient (%)
Gender	
Male	37(42)
Female	51(58)
Age at surgery (years)	
≥ 65	45(51)
< 65	43(49)
Localization	
Colon ascendens	35(40)
Colon descendens	18(20)
Colon transversum	30(34)
Colon sigmoideum/rectum	5(6)
Tumor type	
Adenocarcinoma	67(76)
Adenocarcinoma/mucinous	21(24)
Tumor grade	
Well differentiated	3(3)
Moderately differentiated	80(91)
Poorly differentiated	5(6)
Tumor stage (AJCC)	
I	19(21)
II	26(30)
III	21(24)
IV	22(25)
Lymph node metastasis	
Present	34(39)
Absent	54(61)
Liver metastasis	
Present	22(25)
Absent	66(75)
Lymphovascular invasion	
Present	62(70)
Absent	26(30)
Perineural invasion	
Present	66(75)
Absent	22(25)
Tumor size (cm^3^ )	
≥ 33	46(52)
< 33	42(48)
Total	88

*CRC* colorectal, *AJCC* American Joint Committee on Cancer

### Transcription polymerase chain reaction (RT-qPCR)

RNA was isolated by extraction using Trizol reagent (Invitrogen, Carlsbad, CA, USA) following the manufacturer’s protocol. The RNA integrity was evaluated by conventional PCR and agarose gel electrophoresis. RNA (1 µg) was treated with DNase I, RNase-free, (Thermo Scientific, Wilmington, NC, USA) to eliminate genomic DNA contamination. cDNA was synthesized from the specimens as described previously [22]. Quantitative PCR was performed using the SYBR Green® PCR Master Mix (Invitrogen, Carlsbad, CA—USA) and Applied Biosystems 7500 Real-Time PCR System, using the following primers: LIMK1 F 5′-CAAGGGACTGGTTATGGTGGC-3′; LIMK1 R 5′-CCCCGTCACCGATAAAGGTC-3′; CFL-1 F 5′-GATAAGGACTGCCGCTATGC-3′; CFL-1 R 5′-GCTTGATCCCTGTCAGCTTC-3′; SSH1 F 5′-ACACCGAGGAGAATATCTTGC-3′; SSH1 R 5′-TGAACCCACCATCTCCATCAAG-3′; β-Actin F 5′-TACAATGAGCTGCGTGTGG-3′; and β-Actin R 5′-TAGCACAGCCTGGATAGCAA-3′. We performed the reaction using 30 s at 95 °C, 30 s at 60 °C, and 30 s at 72 °C for CFL-1 primers, 30 s at 95 °C, 30 s at 57 °C, and 30 s at 72 °C for SSH1 primers, and 30 s at 95 °C and 45 s at 65 °C for LIMK1 primers, all using 40 cycles [23–25]. Negative template controls were run in all qPCR experiments to evaluate whether reaction was contaminated with exogenous DNA or to detect primer dimers formation. The differential expression of the selected genes was calculated by the 2^−ΔΔCT^ or 2^−ΔCT^ method [26]. Tumor samples were classified into high or low groups according to the median value.

### Immunohistochemistry and pathological analysis

The histological analysis of cancer specimens was conducted according to the guidelines contained in the 8th edition of the Cancer Staging Manual, edited by the American Joint Committee on Cancer, prior to immunohistochemical analysis by pathologists of INCA [27]. The patient cohort selected included cases of all tumor stages and pathological grades (I–IV). Immunohistochemistry analyses of CFL-1, LIMK1, and SSH1 proteins were performed using formalin-fixed paraffin-embedded CRC blocks from 59 patients (49 with primary tumors and 10 with liver metastases) stored in the Division of Pathology of INCA. Briefly, 3-micron slices were de-paraffinized and antigen retrieval was performed in Trilogy Buffer (Cell Marque, Sigma-Aldrich, Rocklin, CA, USA), at 98 °C, using the steam process for 30 min. Endogenous peroxidases were blocked with a NovoLink Max Polymer Detection kit (Leica Microsystems, Wetzlar, Germany) for 5 min. Anti-LIMK1 (Thermo Fisher—#PA5-14938, 1:1400 dilution), anti-SSH1 (Sigma—#HPA019845, 1:600 dilution), and anti-CFL-1 (Cell Signaling Technology—#5175, 1:2500 dilution) antibodies were incubated overnight at 4 °C. After incubation, the post-primary antibody and the polymer (Novolink, Newcastle upon Tyne, United Kingdom) were added and incubated for 30 min, rinsed, and exposed to a solution of diaminobenzidine for 3 min. Next, the samples were dehydrated with alcohol, cleared in xylene, and mounted. Negative controls were acquired using the same protocol described above, with the omission of the primary antibody. Immunoreactivity (IR) was determined under an optical microscope based on evaluation of staining intensity (on a scale of 1–3) and percentage of tumor cells with positive IR staining (0 to 100%) for each antibody on each slide. The value was calculated by a pathologist (unfamiliar with the experimental groups) by multiplying staining intensity by percentage of cells with positive IR. The median score among all patients was used for separation between high and low expression.

### CMS classification and gene expression analysis

We downloaded the RNA-Seq data from human CRC samples (n = 622, TCGA-COAD, and TCGA-READ) and normal colorectal samples (n = 51) databases available through the Cancer Genome Atlas (TCGA) project using the R package TCGA Biolinks [28]. Patients were selected when they had RNA-Seq data available online through the TCGA Biolinks package, and the exclusion criterion was not having been classified with the CRC molecular subtype classifier tool [2] or not having the staging described in the clinical-pathological information table. Differences in CFL-1, SSH1, and LIMK1 gene expression among the CMS groups were evaluated after samples were classified using the CRC molecular subtype classifier [2]. We also analyzed gene expression according to the tumor stages of the samples when clinicopathological information was available. Correlations between gene pairs were evaluated as well as their impact on patient survival. All analyses were performed in the R environment, and plots were constructed using the ggplot2 package [28].

### Statistical analysis

Patient cohort groups were analyzed using P values calculated from Student’s t-test where two groups were compared. For comparison between more than two groups, ANOVA followed by the Bonferroni post-test were used. The association between CFL-1, SSH1, and LIMK1 expression and clinicopathological parameters was analyzed by χ^2^ or Fisher’s exact tests. Data were analyzed using GraphPad Prism 5.0 (GraphPad Software, San Diego, CA, USA). Lymph node metastasis risk was analyzed using the binary logistic regression method, using univariate and multivariate analysis. Data were analyzed using SPSS version 20.0 software (SPSS; Chicago, IL, USA). Differences in expression levels from TCGA data were analyzed using the Kruskal–Wallis test to compare differences between CMSs and tumor stages. This was followed by post-hoc pairwise comparisons using the Dunn’s test to determine significant differences between each subgroup. The correlation between CFL-1 and SSH1 or LIMK1 was evaluated using the Spearman correlation coefficient test. Overall survival curves were plotted by the Kaplan–Meier method using the R packages for survival analysis “survival” [29], “survminer”, and the log-rank test was used to compare survival estimates across different groups. Hazard ratios (HRs) were adjusted using Cox regression, with a 95% confidence interval. To overall survival analysis, samples expressing the gene above or equal the median were classified as high expression and the others as low expression. Differences were considered statistically significant when *P < 0.05, **P < 0.01, ***P < 0.001, and ****P < 0.0001.

## Results

### Overexpression of LIMK1 predicts worse overall survival in CRC patients

**Fig. 1:**
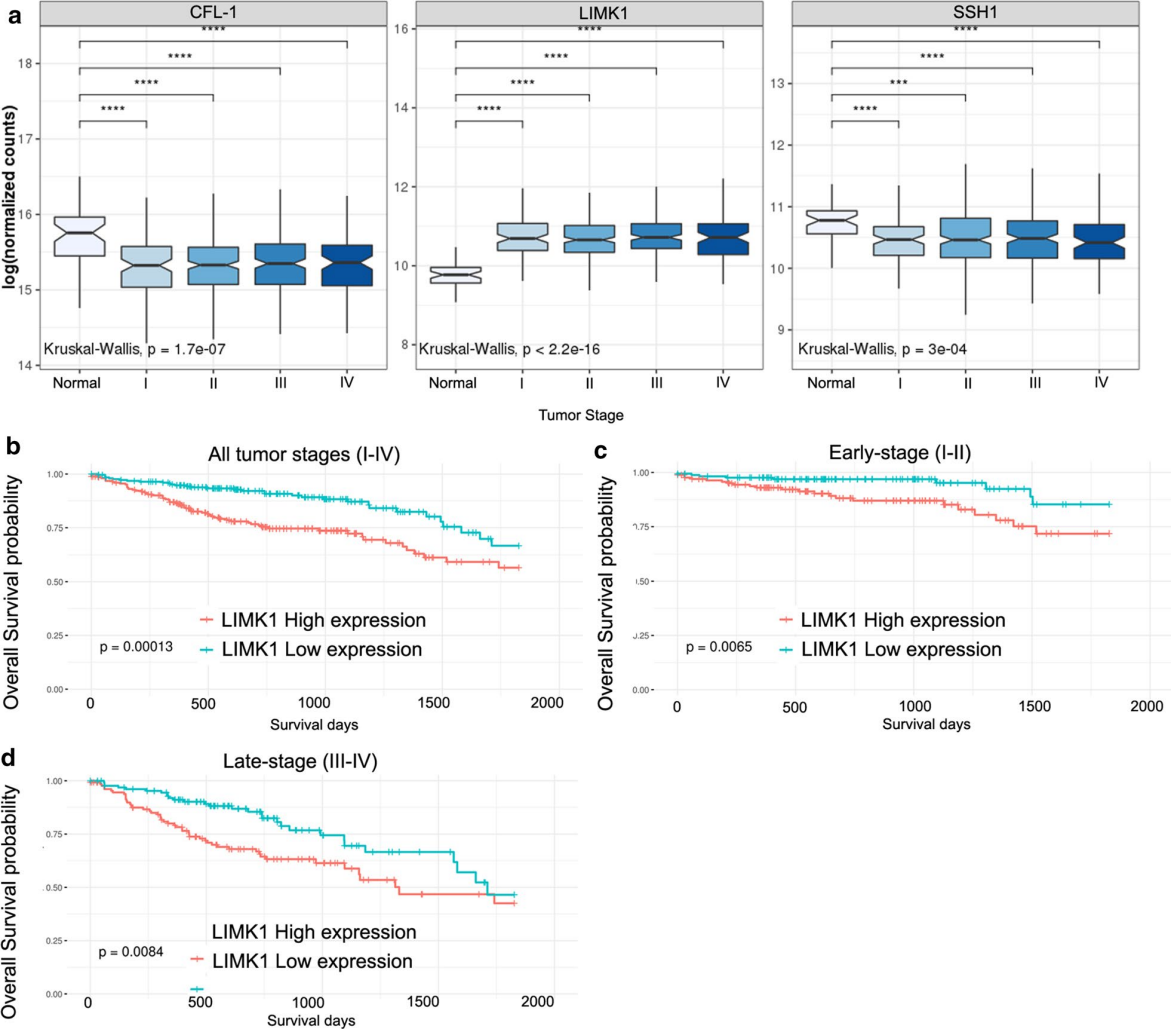
CFL-1, LIMK1, and SSH1 mRNA levels and prognostic value analysis in CRC tissues according to RNA-Seq data from TCGA Data Bank. **a** Expression of CFL-1, LIMK1, and SSH1 in normal colon tissues (n = 51) and according to tumor stage of CRC (n = 599). Kaplan–Meier curves depicting the overall survival (5 years) was generated based on the LIMK1 expression in CRC tissues using TCGA Data Bank. The log-rank test was used to analyze differences in survival curves between the groups. Samples expressing the gene above or equal the median were classified as high expression and the others as low expression. LIMK1 expression was evaluated in **b** all tumor stages I–IV (high n = 266; low n = 264), **c** early stage I–II (high n = 174; low n = 173), **d** late stage III–IV (high n = 137; low n = 136)

To investigate the role that CFL-1 and its regulators, LIMK1 and SSH1, play in human CRC we analyzed the mRNA expression patterns of their coding genes using an in-silico analysis of RNAseq data from patients with CRC (TCGA database). This data comprised of both normal (n = 51) and tumor (n = 622) samples. First, we compared the gene expression levels of each gene among different tumor stages (I (n = 105), II (n = 227), III (n = 179), and IV (n = 88). Twenty-three samples were excluded from this analysis due to a lack of staging information. LIMK1 expression was upregulated (P < 0.0001) whereas CFL-1 and SSH1 were downregulated (P < 0.0001) in all tumor stages when compared with normal tissues (Fig. 1a). However, no significant differences were observed comparing expression levels of each individual gene among different tumor stages (P > 0.05). Since LIMK1 was overexpressed in all tumor stages, we analyzed the impact of these overexpression on overall survival of the patients by Kaplan-Meier curves analysis using TCGA database. Patients were separated according to all tumor stages (I-IV) (Fig. 1b), early stage (I-II) (Fig. 1c), and late-stage (III-IV) (Fig. 1d). A significant worse overall survival rate was observed for patients with high levels of LIMK1 in all tumor stages, early stage, and late stage (P < 0.001, P < 0.01, and P < 0.01, respectively), indicating that high level LIMK1 was an unfavorable prognostic indicator in patients with CRC. However, there were no significant differences in overall survival among subgroups of patients and expression levels of SSH1 (Additional file 1: Figure S1A-B) and to CFL-1 in early stages (Additional file 2: Figure S2A). Patients with low levels of CFL-1 (late-stage III–IV) exhibited lower overall survival (P < 0.05) (Additional file 2: Figure S2B).

### Overexpression of LIMK1 is associated positive lymph node metastasis in CRC patients

**Fig. 2:**
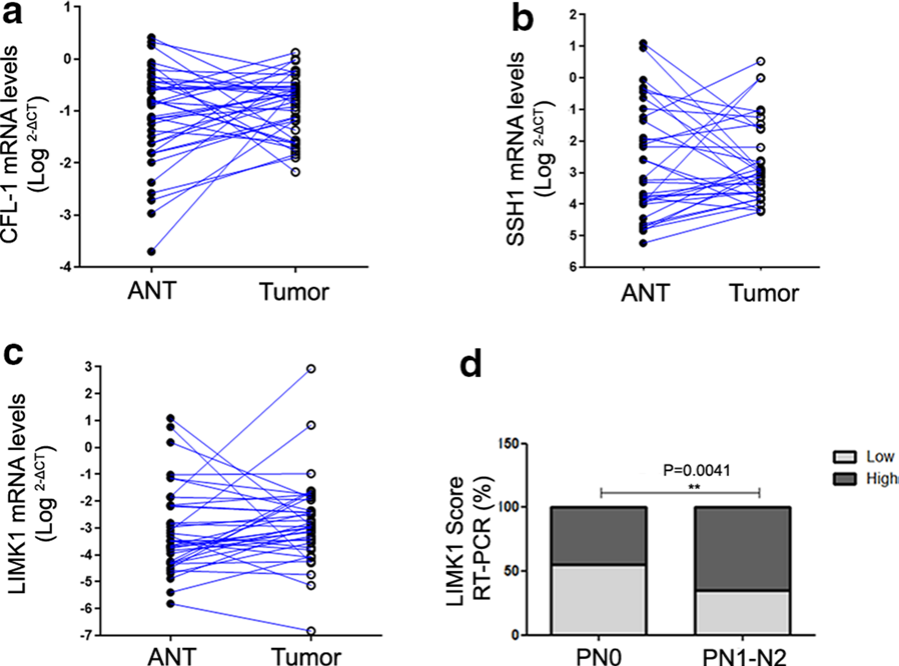
Analysis of CFL-1, LIMK1, and SSH1 mRNA levels and their correlation with clinical features in a clinical cohort. Expression levels were compared using CRC tissues paired their correspondent adjacent normal tissues (ANT) for **a** CFL-1 (n = 38), **b** SSH1 (n = 34) and **c** LIMK1 (n = 37), using RT-qPCR analysis. **d** Association of LIMK1 expression levels with lymph node metastasis. The P values were derived from the χ^2^ test. *P < 0.05; **P < 0.01; ***P < 0.001 and ****P < 0.0001

To confirm the results obtained by bioinformatic analysis, we evaluated CFL-1, LIMK1, and SSH1 mRNA levels by RT-qPCR using tumor tissues from patients with CRC paired with adjacent normal tissues in a clinical cohort. Heterogeneous expression for CFL-1, SSH1 and LIMK1 were detected when tumor tissues were paired with adjacent normal samples (Fig. 2a–c). However, when we separate tumor samples in groups with high or low expression according to the median value obtained by RT-qPCR analysis, only those with higher levels of LIMK1 were associated with lymph node metastasis (N1–N2) (χ^2^, 8.081; P < 0.005) (Fig. 2d). These results suggest that high mRNA level of LIMK1 is associated with positive regional lymph node metastasis in CRC patients.

### CFL-1 and SSH1 protein levels are associated with positive lymph node metastasis in CRC patients

**Fig. 3:**
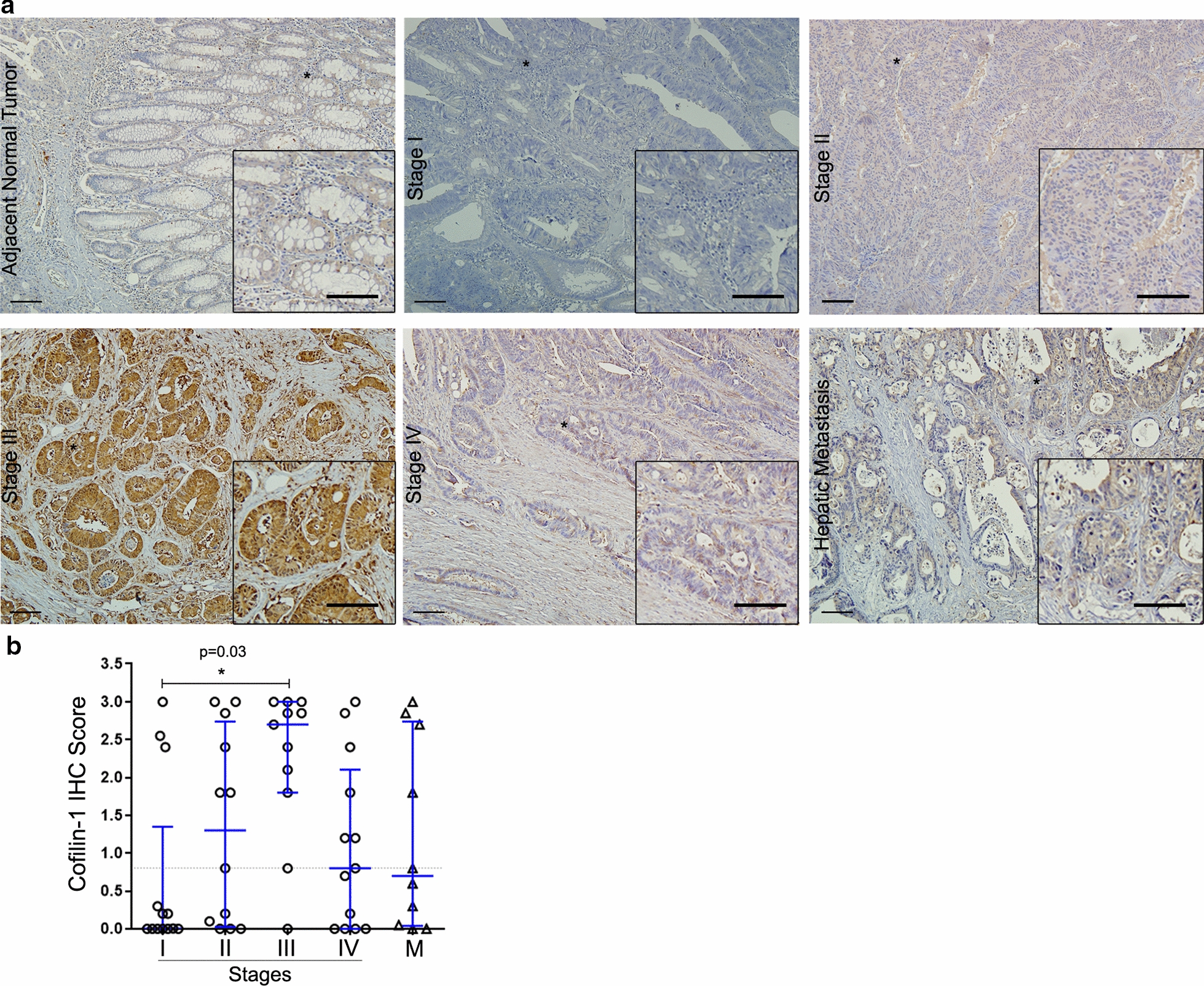
Association between CFL-1 protein levels and local dissemination in CRC patients. **a** Representative IHC images for CFL-1 staining in adjacent normal tissues, in tumor stages (I–IV), and in hepatic metastatic lesions. Scale bar = 100 µm. **b** Quantification of the IHC score data; this value was calculated using the intensity × area of staining. The P value was derived from the paired Student’s t-test (*P < 0.05). IHC and immunohistochemistry of 49 primary tumors (stages I, n = 13; II, n = 12; III, n = 11; and IV, n = 13), and 10 hepatic metastases

We also explored the protein expression patterns in 43 normal colorectal tissues, 49 primary tumors (stages I–IV), and 10 liver metastases by immunohistochemistry (IHC) analysis. CRC samples displayed CFL-1 immunostaining localized in the cytoplasm and membrane, a small number of cells also exhibited nuclear localization. Adjacent normal epithelial tissues displayed either membrane and/or cytoplasmic CFL-1 in the glands of the intestinal crypt (Fig. 3a). Quantitative analysis of CFL-1 staining revealed a higher level (2.7 median score) of CFL-1 in stage III CRC. Stage I patients’ tissues had low-level expression of CFL-1, and exhibited scores of less than 1 (10/13 cases). In patients in stages II, IV, and in liver metastases tissues, CFL-1 expression varied widely, although some isolated cells clearly showed positive staining, even in tissues with low expression levels (Fig. 3b).

To further analyze the association between the data of CFL-1 obtained by IHC score with clinicopathological parameters, we separate tumor samples into high or low expression groups according to the median value obtained in the IHC score. CFL-1 score was significantly related to depth of invasion and metastatic lymph nodes (P = 0.001 and P = 0.02, respectively) (Table 2). Patients with locally advanced invasion (through the muscularis propria into pericolorectal tissues—T3), exhibited a higher CFL-1 score (81.3%). Furthermore, patients with lymph node metastasis also exhibited a higher CFL-1 score (73.7%). However, no significant association between sex, age, localization, stage, lymphovascular and perineural invasion, liver metastasis, tumor size, tumor type, and tumor grade with CFL-1 IHC score was identified (Table 2).

**Table 2:** Association between CFL-1 IHC score and clinicopathological features of CRC patients

Features	n	CFL-1 IHC score, n (%)		χ^2^ value	P-value^a^
		Low	High		
Gender					
Male	23	10(43.4)	13(56.6)	0.208	0.648
Female	26	13(50)	13(50)		
Age at surgery (years)					
≥ 65	27	11(40.7)	16(59.3)	0.927	0.335
< 65	22	12(54.5)	10(45.5)		
Localization					
Colon ascendens	14	3(21.4)	11(78.6)	6.381	0.09
Colon descendens	10	5(50)	5(50)		
Colon transversum	3	1(33.3)	2(66.7)		
Colon sigmoideum/rectum	22	14(63.6)	8(36.4)		
Tumor type					
Adenocarcinoma	40	20(50)	20(50)	0.819	0.364
Adenocarcinoma/mucinous	9	3(33.3)	6(66.7)		
Tumor grade					
Well differentiated	1	1 (100)	0	1.355	0.508
Moderately differentiated	45	21(46.6)	24(53.4)		
Poorly differentiated	3	1(33.3)	2(66.7)		
T stage (AJCC)					
T1/2	15	10(66.7)	5(33.3)	7.985	0.001**
T3	16	3(18.7)	13(81.3)		
T4	18	10(55.5)	8(44.5)		
Lymph node metastasis					
Present	19	5(26.3)	14(73.7)	5.299	0.02*
Absent	30	18(60)	12(40)		
Liver metastasis					
Present	13	7(53.8)	6(46.2)	0.339	0.560
Absent	36	16(44.4)	20(55.6)		
Lymphovascular invasion					
Present	13	7(53.8)	6(46.2)	0.339	0.560
Absent	36	16(44.4)	20(55.6)		
Perineural invasion					
Present	11	6(54.5)	5(45.6)	0.329	0.565
Absent	38	17(44.7)	21(55.3)		
Tumor size (cm^3^ )					
≥ 33	24	10(41.7)	14(58.3)	0.525	0.468
< 33	25	13(52)	12(48)		

^a^P value obtained from χ^2^ test

*CRC* colorectal cancer, *AJCC* American Joint Committee on Cancer

**Fig. 4:**
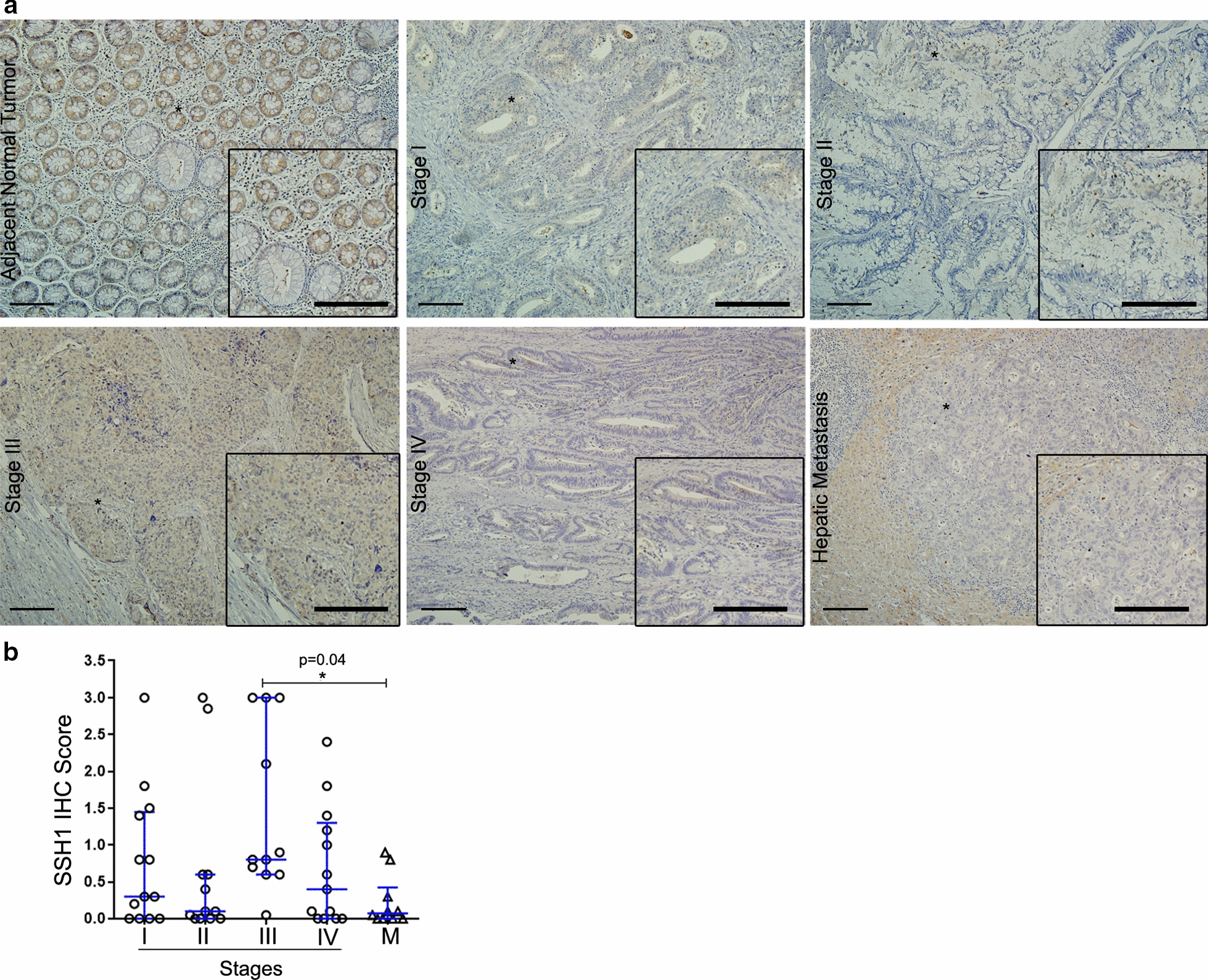
Association between SSH-1 protein levels and lymph node metastasis potential in CRC patients. **a** Representative IHC images for SSH-1 staining in adjacent normal tissues, tumor stages (I–IV), and hepatic metastatic lesions. Scale bar = 100 µm. **b** Quantification of the IHC score data; this value was calculated using the intensity × area of staining. The P value was derived from the paired Student’s t-test (*P < 0.05). IHC, immunohistochemistry of 49 primary tumors (stages I, n = 13; II, n = 12; III, n = 11; and IV, n = 13), and 10 hepatic metastases

Similarly, IHC staining for SSH1 displayed localization in the cytoplasm and membrane in the normal adjacent epithelial layer. IHC quantification revealed high levels (0.8 median score) of SSH1 in stage III tumor tissues, while tissues in stages I, II, IV and in metastatic tissue revealed weak focally positive staining intensity in groups of tumor cells (Fig. 4a, b). When tumor samples were separate into high or low SSH1 expression groups, the SSH1 IHC score was significantly related to lymph node metastasis, similar to CFL-1 data (Table 3). In fact, patients expressing higher levels of SSH1 exhibited a high percentage of T3 grade (68.7%), and lymph node tumor positivity was associated with the SSH1 IHC score (P = 0.02). However, a non-significant association between other clinicopathological parameters and the SSH1 IHC score was observed (Table 3). These data suggest that both CFL-1 and SSH1 play an important role in promoting tumor migration and invasion, leading to dissemination of tumor cells.

**Table 3:** Association between SSH1 IHC score and clinicopathological features of CRC patients

Features	n	SSH1 IHC Score, n (%)		χ^2^ value	P-value^a^
		Low	High		
Gender					
Male	23	9(39.1)	14(60.9)	1.061	0.303
Female	26	14(53.8)	12(46.2)		
Age at surgery (years)					
≥ 65	27	14(51.8)	13(48.2)	0.582	0.442
< 65	22	9(40.9)	13(59.1)		
Localization					
Colon ascendens	14	6(42.8)	8(57.2)	2.773	0.428
Colon descendens	10	7(70)	3(30)		
Colon transversum	3	1(33.3)	2(66.7)		
Colon sigmoideum/rectum	22	9(40.9)	13(59.1)		
Tumor type					
Adenocarcinoma	40	20(50)	20(50)	0.819	0.364
Adenocarcinoma/mucinous	9	3(33.3)	6(66.7)		
Tumor grade					
Well differentiated	1	1(100)	0	1.355	0.508
Moderately differentiated	45	21(46.6)	24(53.4)		
Poorly differentiated	3	1(33.3)	2(66.7)		
T stage (AJCC)					
T1/2	15	8(53.3)	7(46.7)	2.364	0.306
T3	16	5(31.3)	11(68.7)		
T4	18	10(55.5)	8(44.5)		
Lymph node metastasis					
Present	19	5(26.3)	14(73.7)	5.299	0.02*
Absent	30	18(60)	12(40)		
Liver metastasis					
Present	13	7(53.8)	6(46.2)	0.339	0.560
Absent	36	16(44.4)	20(55.6)		
Lymphovascular invasion					
Present	13	6(46.1)	7(53.9)	0.004	0.947
Absent	36	17(47.3)	19(52.7)		
Perineural invasion					
Present	11	5(45.5)	6(54.5)	0.125	0.910
Absent	38	18(47.4)	20(52.6)		
Tumor size (cm^3^)					
≥ 33	24	12(50)	12(50)	0.177	0.674
< 33	25	11(44)	14(56)		

^a^P value obtained from χ^2^ test

*CRC* colorectal cancer, *AJCC* American Joint Committee on Cancer

**Fig. 5:**
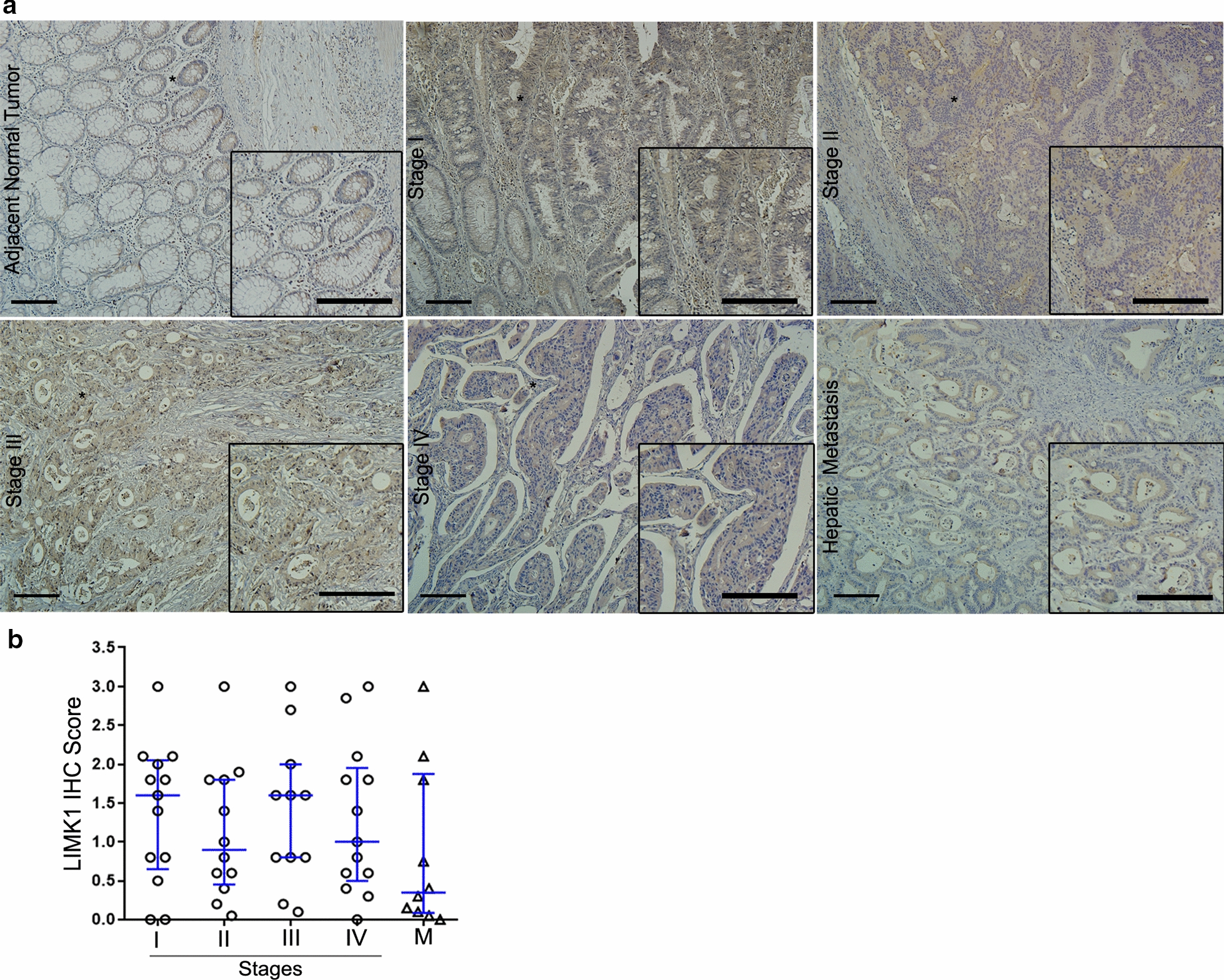
Analysis of LIMK1 protein levels in CRC tissues using IHC. **a** Representative IHC images for LIMK1 staining in adjacent normal tissues, tumor stages (I–IV), and hepatic metastatic lesions. Scale bar = 100 µm. **b** Quantification of the IHC score data; this value was calculated using the intensity × area of staining. IHC and immunohistochemistry of 49 primary tumors (stages I, n = 13; II, n = 12; III, n = 11; and IV, n = 13), and 10 hepatic metastases

LIMK1 immunostaining also occurred in the glands of the intestinal crypt with cytoplasmic localization in adjacent normal tissues. IHC quantitative analysis of LIMK1 did not reach statistical significance because the immunoreactivity levels varied widely among tissue samples (Fig. 5a, b). Furthermore, no association was observed between the LIMK1 IHC score with clinicopathological parameters (Table 4).

**Table 4:** Association between LIMK1 IHC score and clinicopathological features of CRC patients

Features	n	LIMK1 IHC score, n (%)		χ^2^ value	P-value^a^
		Low	High		
Gender					
Male	23	11(47.8)	12(52.2)	0.023	0.879
Female	26	13(50)	13(50)		
Age at surgery (years)					
≥ 65	27	13(48.1)	14(51.9)	0.016	0.897
< 65	22	11(50)	11(50)		
Localization					
Colon ascendens	14	5(35.7)	9(64.3)	5.761	0.123
Colon descendens	10	5(50)	5(50)		
Colon transversum	3	0	3(100)		
Colon sigmoideum/rectum	22	14(63.6)	8(36.4)		
Tumor type					
Adenocarcinoma	40	21(52.5)	19(47.5)	1.080	0.298
Adenocarcinoma/mucinous	9	3(33.3)	6(66.7)		
Tumor grade					
Well differentiated	1	1(100)	0	4.003	0.135
Moderately differentiated	45	23(51.1)	22(48.9)		
Poorly differentiated	3	0	3(100)		
T stage (AJCC)					
T1/2	15	7(46.7)	8(53.3)	0.046	0.977
T3	16	8(50)	8(50)		
T4	18	9(50)	9(50)		
Lymph node metastasis				0.032	0.857
Present	19	9(47.4)	10(52.6)		
Absent	30	15(50)	15(50)		
Liver metastasis					
Present	13	7(53.9)	6(46.1)	0.167	0.682
Absent	36	17(47.2)	19(52.8)		
Lymphovascular invasion					
Present	13	6(46.1)	7(53.9)	0.056	0.812
Absent	36	18(50)	18(50)		
Perineural invasion					
Present	11	6(54.5)	5(45.5)	0.012	0.910
Absent	38	20(52.6)	18(47.4)		
Tumor size (cm^3^)					
≥ 33	24	13(54.1)	11(45.9)	0.506	0.476
< 33	25	11(44)	14(56)		

^a^P value obtained from χ^2^ test

*CRC* colorectal cancer, *AJCC* American Joint Committee on Cancer

To address whether CFL-1 and SSH1 protein levels could be prognostic factors for lymph node metastasis, we performed univariate and multivariate binary logistic regression analyses. In univariate analysis, parameters such as T grade (P = 0.011), lymphovascular invasion (P = 0.012), and high levels of CFL-1 and SSH1 (P = 0.0125) were significantly correlated with lymph node metastasis risk (Table 5). However, multivariate analysis did not support high levels of CFL-1 and SSH1 as independent prognostic factors for CRC (Additional file 3: Table S1). These results suggest that between the clinicopathological factors, depth of invasion, and lymphovascular invasion, the protein levels of CFL-1 and SSH1 could be important factors in predicting lymph node metastases and local advanced disease in CRC.

**Table 5:** Univariate binary logistic regression analysis for the lymph node metastatic risk

Parameter	n	Odds ratio (OR)	95% confidence interval	P value
Gender				
Female	26	0.485	0.151–1.558	0.224
Male	23	1		
Age at surgery (years)				
< 65	23	0.727	0.228–2.316	0.590
≥ 65	26	1		
Tumor size (cm^3^)				
< 33	25	0.788	0.249–2.490	0.684
≥ 33	24			
Localization				
Colon	27	1.203	0.377–3.835	0.755
Colon sigmoideum/rectum	22	1		
Tumor type				
Adenocarcinoma	40	0.241	0.052–1.118	0.069
Mucinous	9	1		
Tumor grade				
Moderately-well	46	0.293	0.025–3.479	0.331
Poor	3	1		
T grade				
T1/2	15	0.063	0.007–0.538	*0.011*
T3/4	34	1		
Lymphovascular invasion				
Absent	36	0.171	0.043–0.683	*0.012*
Present	13	1		
Perineural invasion				
Absent	38	0.264	0.065–1.076	0.063
Present	11	1		
Cofilin-1 IHC score				
Low	23	0.238	0.068–0.836	*0.025*
High	26	1		
SSH1 IHC score				
Low	23	0.238	0.068–0.836	*0.025*
High	26	1		
LIMK1 IHC score				
Low	24	0.900	0.285–2.843	0.858
High	25	1		

### LIMK1 and SSH1 are differentially expressed among the consensus molecular subtypes (CMS)

**Fig. 6:**
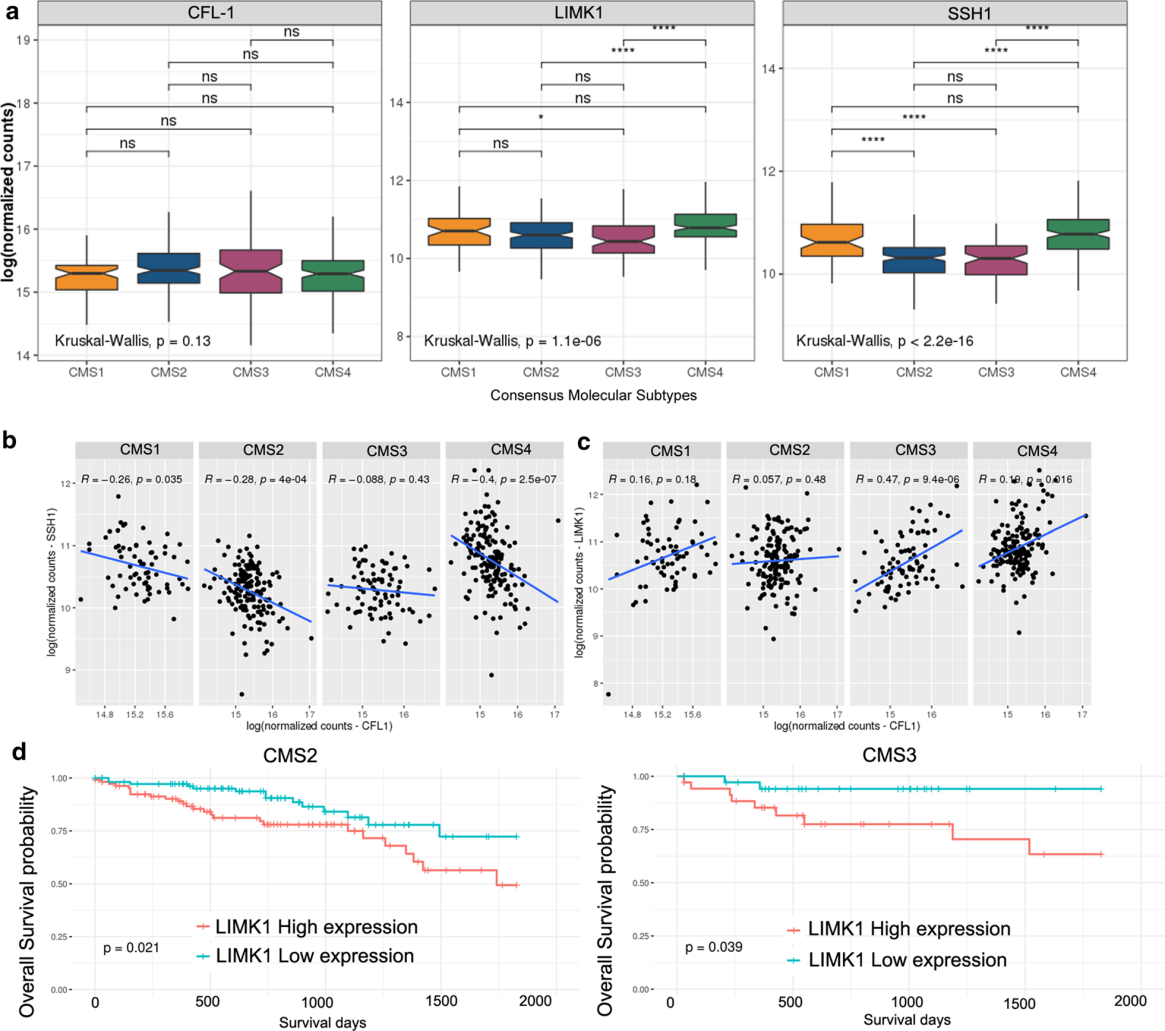
CFL-1, LIMK1 and SSH1 expression, correlation and prognostic value according to the CMS classification using RNA-Seq data from TCGA Data Bank. **a** Tumor tissues were grouped according to the CMS classification (n = 468), and CFL-1, LIMK1, and SSH1 mRNA levels in each subtype were plotted. Kruskal Wallis followed by Dunn’s test were used to compare rank means between each subgroup. Asterisks indicate the padj: *P < 0.05; **P < 0.01; ***P < 0.001 and ****P < 0.0001. Not significant (n.s.). **b** Correlation between CFL-1 and SSH1 or **c** LIMK1 mRNA levels according to CMS classification. The P values and R coefficients were derived from the Spearman correlation test. **d** Kaplan–Meier curves depicting the overall survival (5 years) of CRC patients stratified based on the LIMK1 expression levels according to CMS2 canonical (high n = 113; low n = 112), and CMS3 metabolic subtypes (high n = 36; low n = 36). Samples expressing the gene above or equal the median were classified as high expression and the others as low expression. The P values were derived from the log-rank test, and are indicated. CMS1 immune, CMS2 canonical, CMS3 metabolic, and CMS4 mesenchymal

Next, we sought to determine how CFL-1 and its regulators, LIMK1 and SSH1, are expressed and distributed according to the CMS classification. RNA-Seq data of CRC patients from the TCGA database were used to classify each sample according to the CMS system: 68 samples were classified as CMS1, 155 as CMS2, 84 as CMS3, and 161 as CMS4. One hundred fifty-four samples were not classified. LIMK1 and SSH1 genes, but not CFL-1 gene showed significant differential expression among the CMSs [Kruskal–Wallis P value < 0.05 for LIMK1 and SSH1and P value > 0.05, to CFL-1 (Fig. 6a)]. Notably, LIMK1 expression was highest in CMS4 (median of 10.78) compared to CMS2 and CMS3 (median of 10.60 and 10.43 respectively, P < 0.0001 for both), but was similar to CMS1. In addition, the expression level of SSH1 was similar in CMS2 and CMS3 (median of 10.31 and 10.30 respectively), while CMS1 (median of 10.61) significantly express more SSH1 when compared with CMS2 and CMS3 (P < 0.0001 for both). CMS4 (median of 10.77) exhibited also higher levels of SSH1 expression when compared to the CMS3 and CMS2 subtypes (P < 0.0001 for both). These results revealed that LIMK1 and SSH1 genes are upregulated in immune and mesenchymal subtypes, suggesting a distinct role in the actin dynamic regulation during tumor progression of CRC.

Finally, we analyze the correlation between CFL-1 levels and the regulators SSH1 or LIMK1 according to the CMS classification. We observed that all correlations between CFL-1 and SSH1 mRNA levels were negative (Fig. 6b). In addition, the CMS4 subtype exhibited the highest correlation value (P < 0.0001 and R = − 0.4) among the subtypes. CMS1 (P < 0.05) and CMS2 (P < 0.0001) also exhibited significant correlation between CFL-1 and SSH1 mRNA levels (Fig. 6b). In contrast, all correlations between CFL-1 and LIMK1 mRNA levels were positive. Furthermore, CMS3 and CMS4 exhibited significant correlations between CFL-1 and LIMK1 mRNA levels (P < 0.0001 for CMS3; P < 0.05 for CMS4), and CMS3 exhibited the highest correlation value (R = 0.47) among the subtypes (Fig. 6c). Interestingly, high levels of LIMK1 expression were significantly correlated with lower overall survival rates in the CMS2 and CMS3 subtypes (P < 0.05, Fig. 6d). These results revealed that overexpression of LIMK1 could be useful to detect patients with poor prognosis in CMS2 and CMS3 subtypes. However, there were no significant differences in overall survival among CMSs expression levels of CFL-1 and SSH1 (Additional file 4: Figure S3 and Additional file 5: Figure S4).

## Discussion

Invasion of tumor cells is a key trait of the aggressive phenotype of cancers, and greatly affects the survival of patients due to metastatic outgrowth of disseminated tumor cells [4]. There is considerable evidence indicating that CFL-1 and its regulators, LIMK1 and SSH1, are convergent points of cell signaling networks that play a crucial role in modulating actin cytoskeleton reorganization during malignant progression [8, 30]. However, the clinical significance of these proteins in CRC is not yet fully understood.

We found that LIMK1 mRNA was upregulated in all tumor stages, and patients with high levels exhibited lower overall survival. Although tumor tissue data from our clinical cohort showed heterogeneous expression of LIMK1 mRNA, positive regional lymph node metastasis were positively associated with high levels of LIMK1 expression. These data indicate that LIMK1 could be associated with local invasion during CRC progression and could participate in regional lymph node metastasis. These findings are consistent with other studies in CRC showing that LIMK1 mRNA and protein levels were associated with poor prognosis, including depth of invasion, lymph node metastasis, tumor stage, distal metastases, and reduced survival rate [19, 31, 32]. The mechanism by which LIMK1 can influence poor survival in CRC patients is not known. However, it is possible to suggest that LIMK1 could coordinate proliferation and dissemination of tumor cells by both microtubule disassembly and actin polymerization [33, 34]. Reinforcing this suggestion, it was reported that LIMK1 is crucial for invasiveness and metastatic activity, and regulating the phosphorylation of CFL-1 during mitosis contributes to appropriate cytokinesis, proliferation, and extension of the tumor [34, 35].

Regarding the clinical and biological significance of CFL-1 and SSH1 in CRC, we found that CFL-1 and SSH1 mRNA levels were downregulated in all tumor stages when analyzed by bioinformatic. Indeed, in our clinical cohort, some patients showed low expression of CFL-1 and SSH1 mRNA. Conversely, the upregulation of SSH1 mRNA levels has been observed in CRC tumor tissues when compared with control tissues, and was also associated with tumor stage, lymph node metastasis, and poor prognosis [21]. In addition, SSH1 upregulation was also observed in breast and pancreatic tumors. In breast tumors, SSH1 upregulation was associated with increased metastasis and mortality [15, 36]. In contrast with our data, CFL-1 was upregulated in another small cohort (30 patients) of CRC patients; however, no correlation was found between CFL-1 mRNA levels and clinicopathological features of patients [20]. Interestingly, a study demonstrated progressively reduced levels of phosphorylation, but not mRNA expression, of CFL-1 in CRC patient tissues [17]. Indeed, the low expression levels of CFL-1 and SSH1 in CRC may be explained by the number of patients in each study and particularly the level of methylation in the promoter region that regulates gene expression. In according, we found increased methylation level of the CFL-1 and SSH1 promoter regions in colon and rectum adenocarcinoma tissues from the TCGA database, suggesting that differences in the expression of CFL-1 and SSH1 in CRC may be related to epigenetic modifications (Additional file 6: Figure S5). Supporting this idea, LIMK2, another actin regulator, was downregulated in CRC tissues due to increased promoter methylation [17].

Our IHC profiling identified a direct association between CFL-1 and SSH1 levels with lymph node metastasis in CRC tissues, which was significantly correlated with lymph node metastasis risk by univariate analysis, supporting their role in local tumor dissemination. The results obtained using the multivariate analysis were not significative possibly due to small number of patients enrolled in this study. Further analysis is needed to assess their role in lymph node metastasis risk. However, CFL-1 levels association with dedifferentiation, infiltration depth, pathological staging, and presence of lymph node metastasis have also been found in other cancer types [11–13, 37, 38]. It is possible to speculate that the invasive margin may be region within the tumor that exhibits the highest levels of CFL-1, which increases aggressiveness and leads to local dissemination. However, further investigation is required to determine the relationship between CFL-1 and SSH1 expression levels and their role in different areas of the tumor.

Our bioinformatic analysis showed that the CMS1 and CMS4 subtypes exhibited the highest levels of LIMK1 and SSH1 expression among the analyzed subtypes, while CFL-1 levels were homogeneous according to CMSs. It is important point out that CMS1 and CMS4 subtypes are characterized by an extensive prominent stromal invasion, fibroblast-rich content, angiogenesis, and poor outcome among the CMSs. CMS1 subtype have the worst survival after relapse while CMS4 exhibits the worst prognosis subtype [2]. In agreement with our results, previous reports have shown a relevant association between these proteins and EMT markers [E-cadherin, β-catenin (nuclear), ZEB1, and SNAIL] in CRC tissues [19, 21], indicating their central role during EMT in CRC. We previously demonstrated, using an in vitro model, the dynamic role of CFL-1 and LIMK2 during EMT in CRC [25]. In accordance with these data, studies using bladder, prostate, and gastric models have also correlated CFL-1 and LIMK1 expression pathways with the EMT program [14, 39, 40], as well as tumor initiation and metastatic colonization in breast model [41]. Notably, these gene expression could not be constant throughout the different areas of the tumor probably due to the high intratumor heterogeneity of CRC [2]. One study using CRC tissues showed that tumor buddings exhibited differential gene expression related to the actin cytoskeleton remodeling pathway, including EMT signatures when compared with tumor bulks, and also alternated from CMS2 in the tumor bulk to CMS4 in budding cells [42]. Furthermore, we noticed a significant correlation between CFL-1 and LIMK1/SSH1 expression according to the CMS classification suggesting that the modulation of CFL-1 (activation/non-activation) can be more complex and regulated to different degrees depending on the CMS. Aggressive subtypes, such as mesenchymal (CMS4), may have a higher degree of dependency on CFL-1 regulators, and could impact on higher invasion rates.

Moreover, we observed that high levels of LIMK1 mRNA significantly correlated with lower overall survival in canonical and metabolic subtypes. The major pathway related to the canonical subtype is Wnt signaling. Interestingly, evidence has shown that LIMK1 can bind β-catenin protein and translocate into the nucleus, enhancing CRC cell metastasis [18]. Regarding metabolic LIMK1 functions, some studies have suggested that insulin and high glucose levels may stimulate actin cytoskeleton remodeling, with consequent phosphorylation of LIMK1 mediating GLUT4 translocation to the cell surface and glucose uptake [43–45]. However, further investigation is required to verify the role of LIMK1 in canonical and metabolic subtypes in CRC since most of these studies were evaluated using in vitro models. Here, we used bioinformatics approaches to show the clinical significance of these proteins and their relationship with distinct biological phenotypes, according to the CMS classification. Additionally, since these proteins could mediate the dissemination of tumor cells, their mRNA and correlation analyses may be useful for stratifying patients with potential risk for metastatic disease in each CMS.

## Conclusions

In conclusion, our data suggest that CFL-1 and its regulators LIMK1 and SSH1, are differentially expressed in CRC. Since patients with lymph node metastasis exhibited RNA and/or protein upregulation, these might indicate their crucial role in regional cancer cell dissemination and also might be useful for predicting metastatic disease in CRC. A better and more detailed clinical stratification by CMS, as well as new treatment approaches using these proteins as targets, might provide improvements in therapeutic outcomes for CRC patients, avoiding early tumor dissemination, particularly in immune and mesenchymal subtypes.

## Supplementary Information

**Additional file 1: Figure S1.** Analysis of overall survival according to SSH1 expression according to tumor stage. Kaplan–Meier curves depicting the overall survival using CRC patient’s data from TCGA Data Bank were stratified based on SSH1 expression level according to tumor stage (early/late). (a) All tumor stages I–IV (High n=266; Low n=264), (b) early stage I-II (High n=174; Low n=173 and (c) late stage III-IV (High n=137; Low n=136). Samples expressing the gene above or equal the median were classified as high expression and the others as low expression. The P values were derived from the log-rank test, and are indicated.

**Additional file 2: Figure S2.** Analysis of overall survival according to CFL-1 expression according to tumor stage. Kaplan–Meier curves depicting the overall survival using CRC patient’s data from TCGA Data Bank were stratified based on CFL-1 expression level according to tumor stage (early/late). (a) Early stage I-II (High n=174; Low n=173 and (b) late stage III-IV (High n=137; Low n=136). Samples expressing the gene above or equal the median were classified as high expression and the others as low expression. The P values were derived from the log-rank test, and are indicated.

**Additional file 3: Table S1.** Multivariate analysis for the lymph node metastatic risk.

**Additional file 4: Figure S3.** Analysis of overall survival according to CFL-1 expression in CRC tissues according to CMS classification. Kaplan–Meier curves depicting the overall survival were generated using CRC patient’s data from TCGA Data Bank. (a) CMS1 immune (High n=42; Low n=41), (b) CMS2 canonical (High n=113; Low n=112), (c) CMS3 metabolic (High n=36; Low n=36), and (d) CMS4 mesenchymal (High n=75; Low n=75). The P values were derived from the log-rank test.

**Additional file 5: Figure S4.** Analysis of overall survival according to SSH1 expression in CRC tissues according to CMS classification. Kaplan–Meier curves depicting the overall survival were generated using CRC patient’s data from TCGA Data Bank. (a) CMS1 immune (High n=42; Low n=41), (b) CMS2 canonical (High n=113; Low n=112), (c) CMS3 metabolic (High n=36; Low n=36), and (d) CMS4 mesenchymal (High n=75; Low n=75). The P values were derived from the log-rank test.

**Additional file 6: Figure S5.** Analysis of methylation levels in the promoter region of CFL-1 (a) and SSH1 (b) using the UALCAN approach. Colon adenocarcinoma (COAD) and rectal adenocarcinoma (READ) from the TCGA database were used. *P<0.05; **P<0.01; ***P<0.001 and ****P<0.0001. Not significant (n.s.).

## Abbreviations

ANT: Adjacent normal tissue
AJCC: The American Joint Committee on Cancer
CFL-1: Cofilin-1
CMSs: Consensus Molecular Subtypes
CMS1: Immune
CMS2: Canonical
CMS3: Metabolic
CMS4: Mesenchymal
CRC: Colorectal cancer
EMT: Epithelial–mesenchymal transition
IHC: Immunohistochemistry
LIMK1: LIM domain kinase 1
SSH1: Slingshot protein phosphatase 1
