# Supplementary figures and images for: Cofilin-1, LIMK1 and SSH1 are differentially expressed in locally advanced colorectal cancer and according to consensus molecular subtypes
Source: Cancer Cell Int. 2021 Jan 22;21:69. doi: 10.1186/s12935-021-01770-w (PMC7821653; doi:10.1186/s12935-021-01770-w)

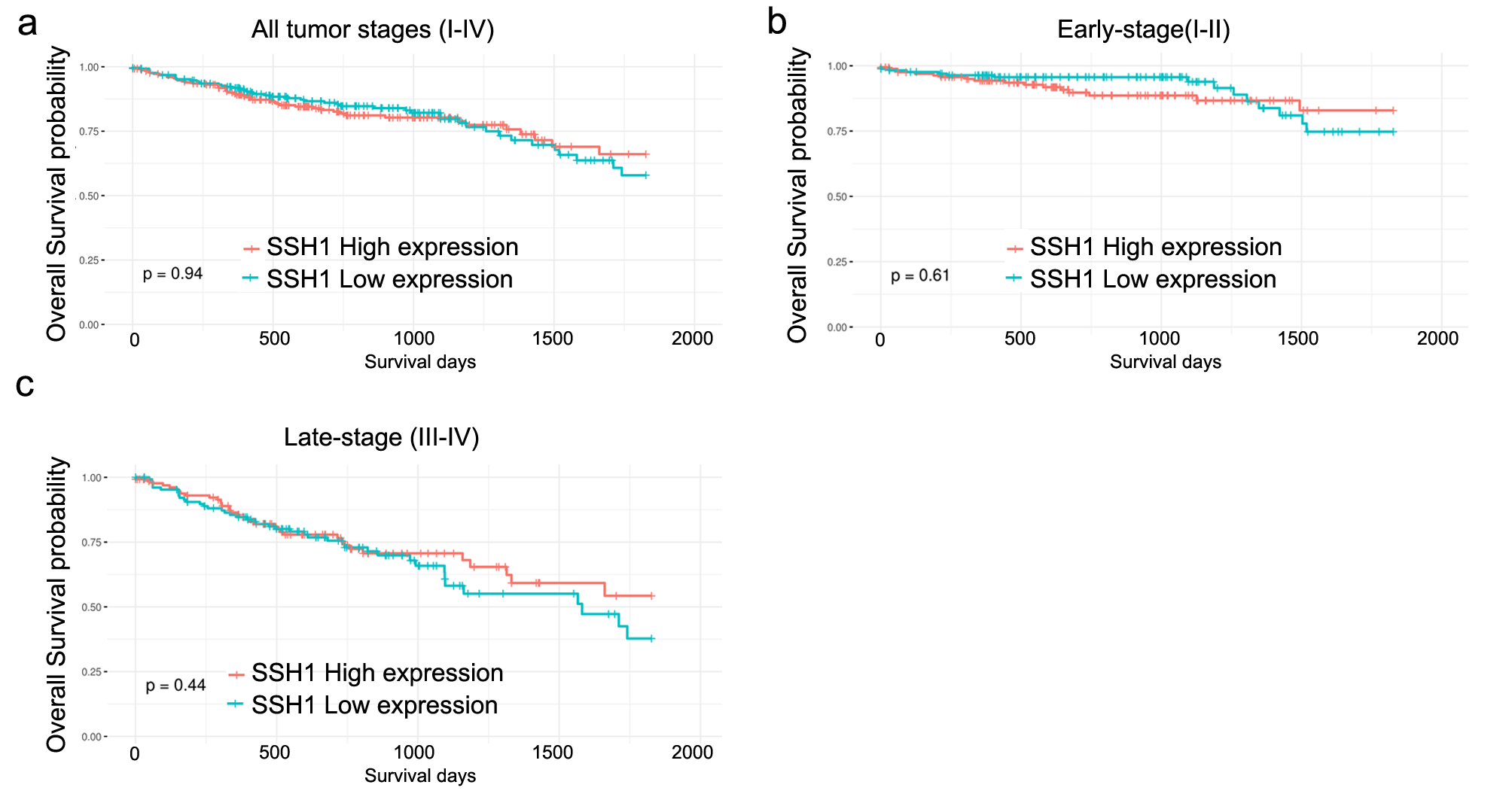

Supplement: Supplementary file 1 — Additional file 1: Figure S1. Analysis of overall survival according to SSH1 expression according to tumor stage. Kaplan–Meier curves depicting the overall survival using CRC patient’s data from TCGA Data Bank were stratified based on SSH1 expression level according to tumor stage (early/late). (a) All tumor stages I–IV (High n=266; Low n=264), (b) early stage I-II (High n=174; Low n=173 and (c) late stage III-IV (High n=137; Low n=136). Samples expressing the gene above or equal the median were classified as high expression and the others as low expression. The P values were derived from the log-rank test, and are indicated. [file 12935_2021_1770_MOESM1_ESM.tif]

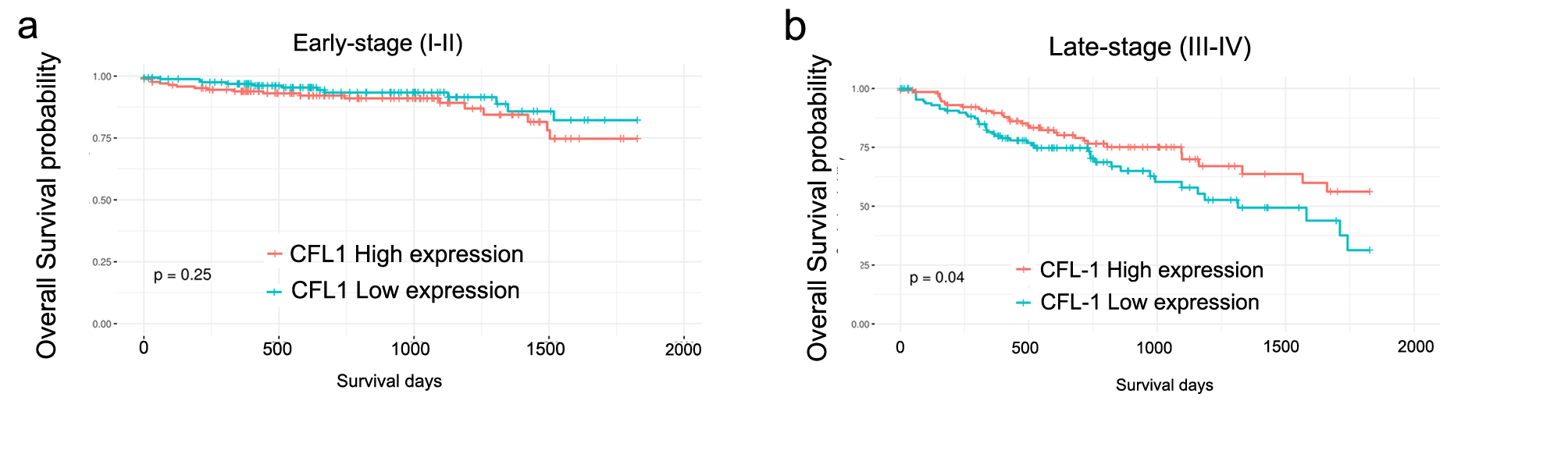

Supplement: Supplementary file 2 — Additional file 2: Figure S2. Analysis of overall survival according to CFL-1 expression according to tumor stage. Kaplan–Meier curves depicting the overall survival using CRC patient’s data from TCGA Data Bank were stratified based on CFL-1 expression level according to tumor stage (early/late). (a) Early stage I-II (High n=174; Low n=173 and (b) late stage III-IV (High n=137; Low n=136). Samples expressing the gene above or equal the median were classified as high expression and the others as low expression. The P values were derived from the log-rank test, and are indicated. [file 12935_2021_1770_MOESM2_ESM.tif]

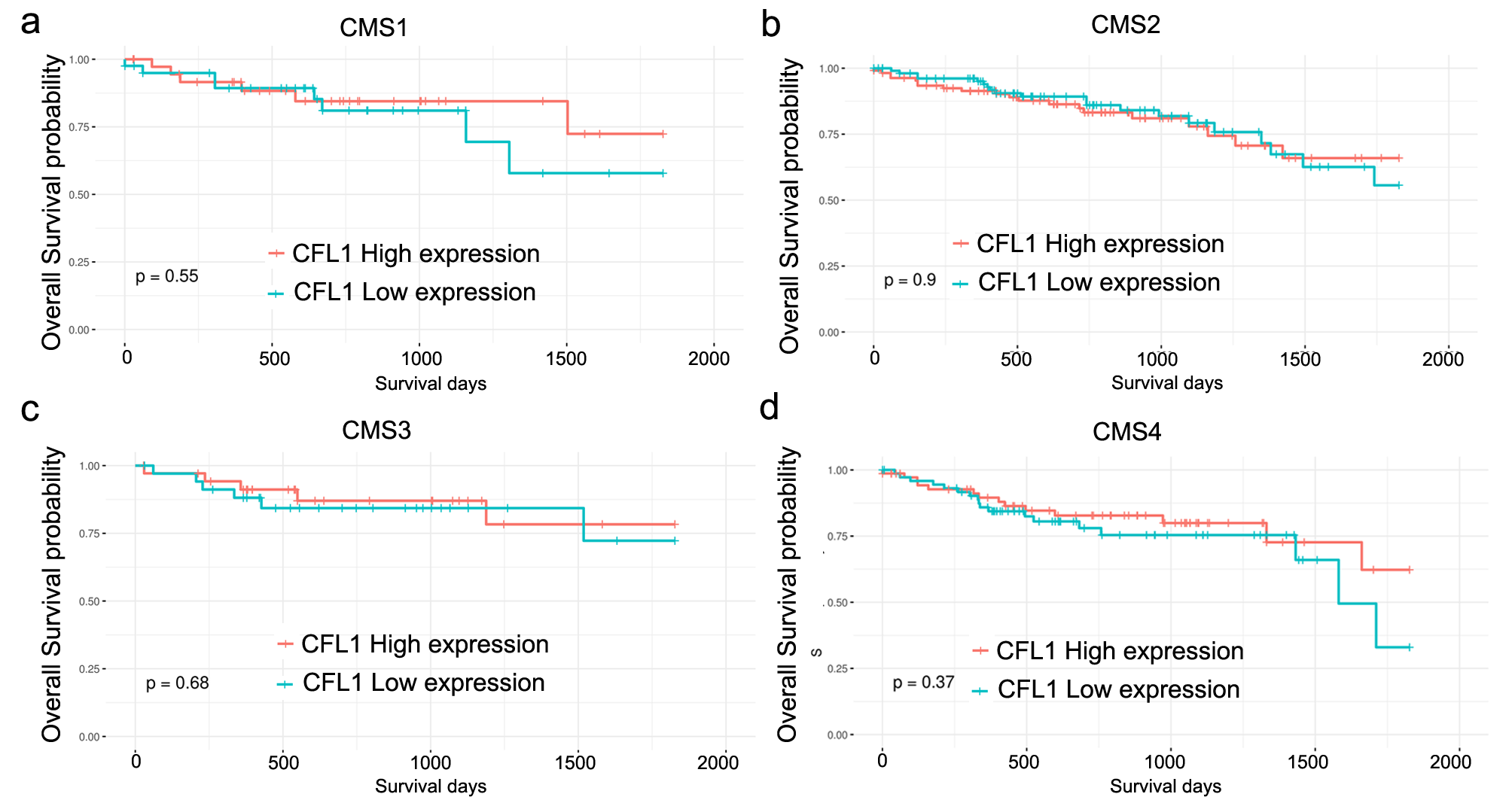

Supplement: Supplementary file 4 — Additional file 4: Figure S3. Analysis of overall survival according to CFL-1 expression in CRC tissues according to CMS classification. Kaplan–Meier curves depicting the overall survival were generated using CRC patient’s data from TCGA Data Bank. (a) CMS1 immune (High n=42; Low n=41), (b) CMS2 canonical (High n=113; Low n=112), (c) CMS3 metabolic (High n=36; Low n=36), and (d) CMS4 mesenchymal (High n=75; Low n=75). The P values were derived from the log-rank test. [file 12935_2021_1770_MOESM4_ESM.tif]

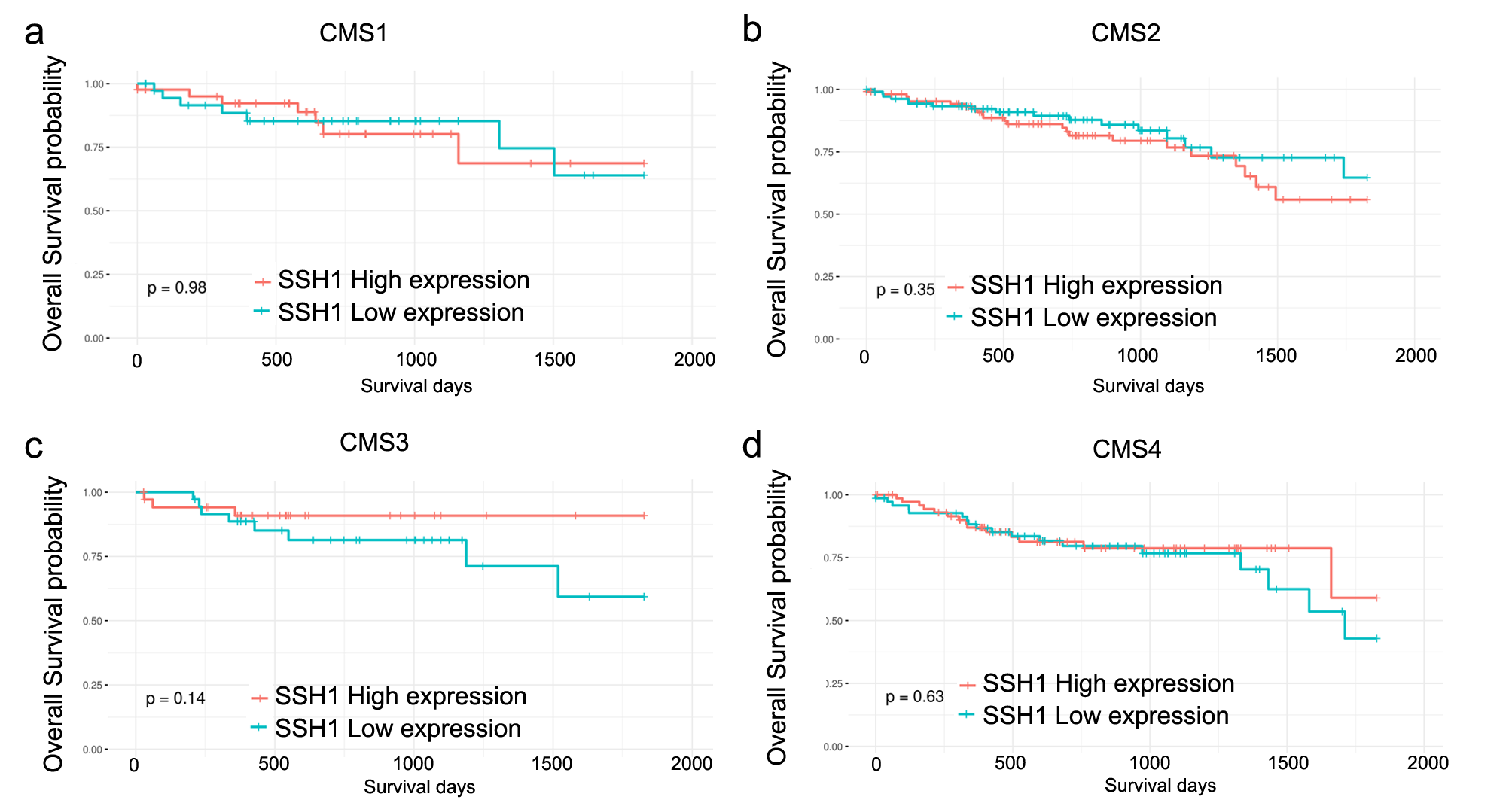

Supplement: Supplementary file 5 — Additional file 5: Figure S4. Analysis of overall survival according to SSH1 expression in CRC tissues according to CMS classification. Kaplan–Meier curves depicting the overall survival were generated using CRC patient’s data from TCGA Data Bank. (a) CMS1 immune (High n=42; Low n=41), (b) CMS2 canonical (High n=113; Low n=112), (c) CMS3 metabolic (High n=36; Low n=36), and (d) CMS4 mesenchymal (High n=75; Low n=75). The P values were derived from the log-rank test. [file 12935_2021_1770_MOESM5_ESM.tif]

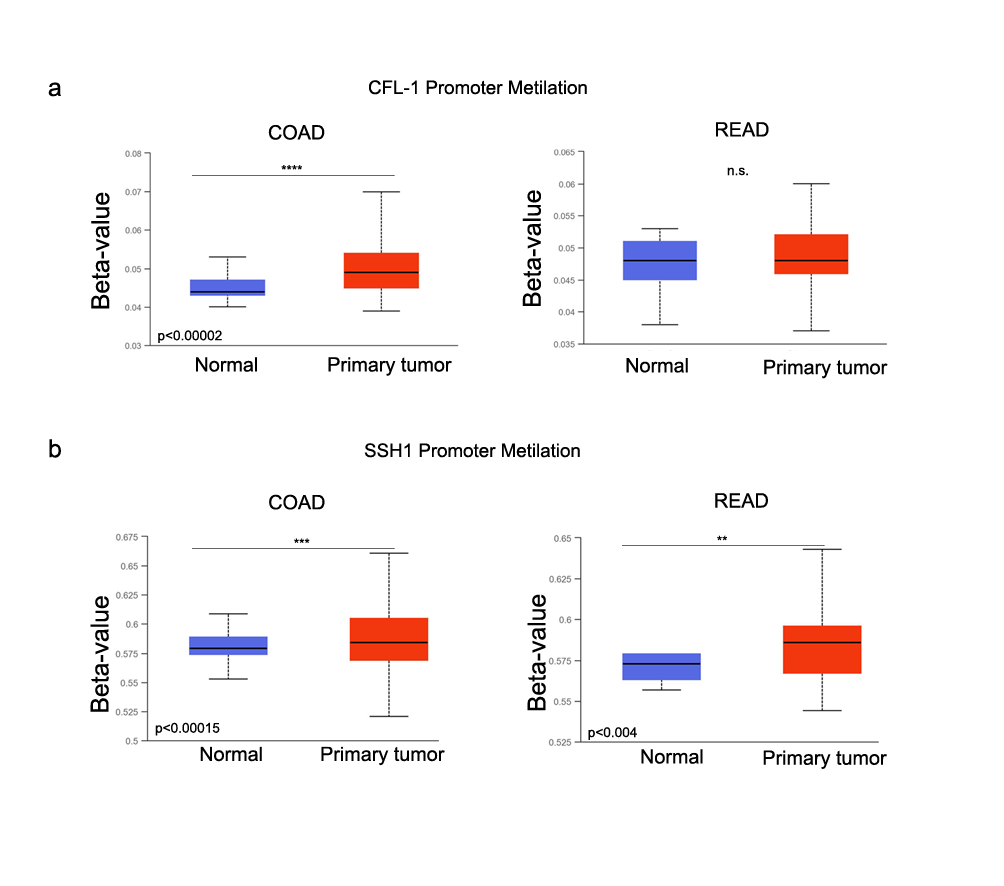

Supplement: Supplementary file 6 — Additional file 6: Figure S5. Analysis of methylation levels in the promoter region of CFL-1 (a) and SSH1 (b) using the UALCAN approach. Colon adenocarcinoma (COAD) and rectal adenocarcinoma (READ) from the TCGA database were used. *P<0.05; **P<0.01; ***P<0.001 and ****P<0.0001. Not significant (n.s.). [file 12935_2021_1770_MOESM6_ESM.tif]
