# Supplementary material for: Cofilin-1, LIMK1 and SSH1 are differentially expressed in locally advanced colorectal cancer and according to consensus molecular subtypes
Source: Cancer Cell Int. 2021 Jan 22;21:69. doi: 10.1186/s12935-021-01770-w (PMC7821653; doi:10.1186/s12935-021-01770-w)
Supplement: Supplementary file 3 — Additional file 3: Table S1. Multivariate analysis for the lymph node metastatic risk. [file 12935_2021_1770_MOESM3_ESM.docx]

| **Table S1.** Multivariate analysis for the lymph node metastatic risk | | | |  |
| --- | --- | --- | --- | --- |
| **Parameter** | **n** | **Odds Ratio (OR)** | **95% confidence interval** | **P value** |
| **CFL-1 IHC Score** |  |  |  |  |
| Low | 23 | 0.220 | 0.033-1.450 | 0.115 |
| High | 26 |  |  |  |
| **SSH1 IHC Score** |  |  |  |  |
| Low | 23 | 0.199 | 0.037-1.060 | 0.059 |
| High | 26 |  |  |  |
| **Tumor type** |  |  |  |  |
| Adenocarcinoma | 40 | 0.428 | 0.047-3.910 | 0.452 |
| Mucinous | 9 |  |  |  |
| **T grade** |  |  |  |  |
| T1/2 | 15 | 0.167 | 0.014-1.998 | 0.158 |
| T3/4 | 34 |  |  |  |
| **Lymphovascular invasion** |  |  |  |  |
| Absent | 36 | 0.128 | 0.017-0.981 | **0.048** |
| Present | 13 |  |  |  |
| **Perineural invasion** |  |  |  |  |
| Absent | 38 | 0.600 | 0.085-4.239 | 0.609 |
| Present | 11 |  |  |  |
